# Protocol for indirect and direct co-culture between human cancer cells and endothelial cells

**DOI:** 10.1016/j.xpro.2023.102177

**Published:** 2023-04-21

**Authors:** Yichen Guo, Bronte Miller, Michael Heim, Ana Gutierrez-Garcia, Renata Jaskula-Sztul, Bin Ren, Mary Kathryn Sewell-Loftin

**Affiliations:** 1 Department of Biomedical Engineering, University of Alabama at Birmingham, Birmingham AL 35294, USA; 2 Department of Surgery, University of Alabama at Birmingham, Birmingham AL 35233, USA; 3 O’Neal Comprehensive Cancer Center, University of Alabama at Birmingham, Birmingham AL 35233, USA

**Keywords:** Biotechnology and Bioengineering, Cancer, Cell Biology, Cell Culture, Cell-based Assays, Microscopy, Molecular Biology, Tissue Engineering

## Abstract

The cross talk between cancer cells and endothelial cells (ECs) within the tumor microenvironment plays a critical role in tumor progression, recurrence, and cancer stemness. Here, we present a protocol containing two *in vitro* approaches to study such interactions. We first describe an indirect co-culture system to study the regulation of stemness markers in cancer cells by secreted factors from ECs. We then detail a direct co-culture system to study juxtracrine communications between the cell types.

For complete details on the use and execution of this protocol, please refer to Sewell-Loftin et al.^1^ and Guo et al.^2^

## Highlights

• An optimized protocol for indirect co-culture between cancer cells and ECs
• RT-qPCR-based analysis of the stemness marker expression following indirect co-culture
• Vasculogenic ring assay for 3D direct co-culture of stromal and cancer cells
• Immunofluorescence staining and imaging for cells cultured in the 3D *in vitro* ring assay

## Abstract (editor highlights)

Publisher’s note: Undertaking any experimental protocol requires adherence to local institutional guidelines for laboratory safety and ethics.

## Graphical abstract

**Figure:**
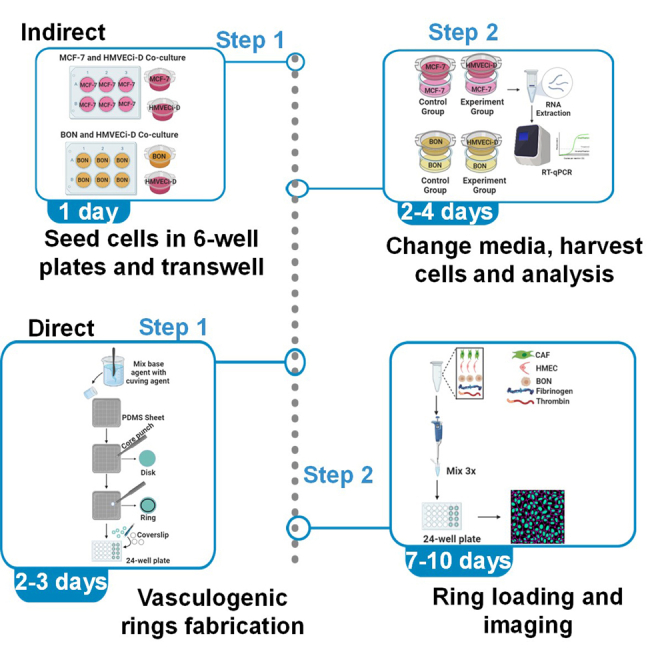

## Before you begin

Overall Objectives: This protocol includes two different *in vitro* methods to co-culture cancer cells and vascular ECs. The first method is an indirect co-culture by transwell; our discussion of the protocol includes how to optimize concentrations of and ratios between cell types to study changes in cancer stem-like cell markers in cancer cell lines. The second method is a direct co-culture by the vasculogenic ring assay, in which cells are embedded in a disk-shaped ECM-based gel. To accomplish this, users fabricate poly(dimethylsiloxane) (PDMS) rings by using concentric core punches on a thin sheet of PDMS. The ring attaches to a glass coverslip; ultimately, this 3D model provides a highly rigorous and repeatable geometry for the 3D *in vitro* models to eliminate geometrical aberrations in casting the ECM-based gels. The assay is referred to as the vasculogenic ring assay, based on its origination to look at vascular growth and the ring-based geometry.^1^ The indirect protocol permits investigation of secreted factors from ECs on the regulation of stemness markers in cancer cells. While the methodology is fairly well established, the analysis of stemness markers in various types of cancer cells shows trends in stemness regulation based solely on ligand signaling. In contrast, the direct co-culture method provides an enhanced model of the tumor microenvironment (TME) where stromal components are adjacent to cancer cells, permitting investigations of juxtracrine signaling through ligand and cell-cell contact pathways. Comparing indirect and direct co-culture models provides a more thorough investigation of regulation cancer cell stem markers. Our results show how the cancer stemness-related markers including CD44, CD36, and ALDH1A1 are differentially regulated in indirect vs. direct co-culture methods. Read carefully and prepare materials and reagents before starting the experiments.

Cell Culture Selection: MCF-7 cells are human breast cancer cells positive for estrogen receptors. BON cells are a pancreatic neuroendocrine tumor (pNET) cell line. These two cancer cells are used for indirect co-culture with HMVECi-D, which were generated from the primary human dermal microvascular endothelial cells (HMVEC-D) transduced with specific SV40 large T antigen and telomerase. These cell lines were selected for the studies based on previous experience in the authors’ labs. Furthermore, MCF-7 breast cancer cells are widely characterized, allowing for comparison of results from the included studies with literature. Expanding to perform studies with the pNET cell line also permitted comparisons of stem marker regulation in multiple cancer types. The HMVECi-D cells are a well-established EC model.^3^ For direct co-culture, an alternative EC line was chosen, HMEC-1 (ATCC, CRL-3243) which is used for 3D TME models to study blood vessel growth. Additionally, immortalized breast cancer-associated fibroblasts (CAFs) were used as a stromal cell in some experiments. All cell lines described in this protocol were immortalized, and passage number was not considered during experiments. Users should refer to specifications for their cell lines of interest to determine if passage number is an important parameter while planning their studies.

### Institutional permissions

The protocols described in this manuscript utilized commercially-available or previously established cell lines only. The protocols may be expanded to use primary cells sourced from either animal models or clinical specimens; readers are reminded to follow all appropriate institutional requirements for the use of such materials.

### Prepare Mammalian cell lines

**Timing: 7 days**

1. Prepare cell culture medium as shown in the tables in the “materials and equipment” section. Incubate all cell lines at 5% CO_2_, 37°C.
2. Prepare MCF-7, BON, HMVECi-D, HMEC-1, and CAF cells for co-culture.
  a. Day 1: Revive all cell lines from liquid nitrogen tank. Culture these cell lines in separate 10 cm dishes with appropriate culture medium.
  b. Day 2: Check cellular quality to make sure the cells are suitable for co-culture. Change medium to remove unattached cells.
    ***Note:*** In this case, “suitable” refers to whether cells have successfully seeded and proliferated following the revival from cryostorage. CAFs and cancer cells often revive faster and may be ready to passage before the EC lines.
  c. Day 4: Passage cells at least once prior to use in indirect co-culture protocols.
    i. To begin, aspirate culture medium and rinse with DPBS to remove cellular debris; wash volumes should be sufficient to cover the surface of the culture flask (∼3–4 mL for 10 cm dish).
    ii. Add 3 mL of trypsin and incubate plates at 37°C for 2–3 min.
    iii. Check dishes on a microscope to determine if cells have detached.
    iv. Add 6 mL of appropriate media to neutralize the trypsin.
    v. Transfer cell suspensions to labeled 15 mL conical tubes for centrifugation.
      ***Note:*** All cell lines utilized in this protocol can be split at 1:3, with passaged cells replated on new 10 cm dishes. Continue the cell culture.
      ***Note:*** Make sure the cells reach 90% confluence before starting the co-culture experiment.
      ***Note:*** Different cell lines require different formulations of trypsin. We recommend that HMEC-1 and CAFs are treated with 0.25% EDTA Trypsin while all other cell lines should be treated with 0.05% EDTA Trypsin. See key resources table for more information.

### Design primers

**Timing: 1 day**

3. Determine genes of interest. Check the gene ID from the IDT bank via the NCBI website.

***Note:*** Be sure to verify the appropriate organisms for your targeted genes (i.e., – *Homo sapiens*, *Mus musculus*, etc.).

4. Search the gene ID in Primer Bank to find the sequence recommended in the bank.
  a. For the primers used in the study below, please see Table 1.

**Table 1:** Primer sets used in this study

Primer	Sequence	Source
CD133	Forward: AGTCGGAAACTGGCAGATAGC Reverse: GGTAGTGTTGTACTGGGCCAAT	NCBI Primer Bank
CD24	Forward: CAGATCCAAGCATCCTGAGCA Reverse: CGTGGTCAATGCAATTCTACTCT	Best et al.^3^
CD36	Forward: CTTTGGCTTAATGAGACTGGGAC Reverse: GCAACAAACATCACCACACCA	NCBI Primer Bank
CD44	Forward: CTGCCGCTTTGCAGGTGTA Reverse: CATTGTGGGCAAGGTGCTATT	NCBI Primer Bank
MYC	Forward: GTCAAGAGGCGAACACACAAC Reverse: TTGGACGGACAGGATGTATGC	NCBI Primer Bank
KLF4	Sequence from IDT	IDT Bank
ALDH1A1	Forward: GCACGCCAGACTTACCTGTC Reverse: CCTCCTCAGTTGCAGGATTAAAG	NCBI Primer Bank
PRKD-1	Sequence from IDT	IDT Bank

***Note:*** In Primer Bank, there are several primer sequence choices for each gene. Check the sequence in NCBI Primer Blast to make sure that the sequence targets the specific gene. It is not recommended to use a sequence that may target other genes.

***Note:*** If the primer sequences are already used in previous publication(s), they can be synthesized based on the published paper. However, it is important to double check the sequence by NCBI Primer Blast.

***Note:*** All the primer sequences listed in Table 1 are validated by NCBI Primer Blast and only target the specific gene of interest.

### Prepare vasculogenic rings

**Timing: 2–3 days**

The vasculogenic ring assay is the protocol for the direct co-culture assay. The platform is composed of a silicone-based polymer ring on a glass coverslip. The model provides reliable geometric confinement for ECM-based 3D tissue culture models.

5. Pouring polydimethylsiloxane (PDMS) sheets.
  ***Note:*** PDMS sheets are cured in 10 × 10 cm square dishes (Fisher 50-190-0277) at room temperature (22°C–27°C). Each sheet is large enough to generate approximately 40 rings (ID = 8 mm, OD = 10 mm). The protocol below is based on 1 sheet and may be scaled accordingly.
  a. Determine the number of sheets needed.
    ***Note:*** Each sheet requires 7 g of PDMS. Users should prepare more than 7 g for each sheet; we recommend mixing 11 g for preparing one sheet as detailed below. The PDMS is viscous, and some of the solution will adhere and remain in the disposable plastic cup. Thus, extra PDMS should be prepared.
  b. Determine the amount of base agent to curing agent with the weight ratio of 10:1. E.g., Mix 1 g Curing Agent with 10 g Base Agent for each PDMS sheet. See Table 2 for details.

**Table 2:** Formulation for PDMS sheet generation

Reagent	Mass needed
Sylgard 184 Curing Agent	1 g
Sylgard 184 Base Agent	10 g
  c. Put a disposable plastic cup on the balance and tare the balance.
  d. Add 1 g Curing Agent to the disposable plastic cup and re-tare the balance.
  e. Carefully and slowly add 10 g Base Agent to Curing Agent in the disposable plastic cup.
    ***Note:*** If you over-pour Base Agent, add extra Curing Agent to maintain the weight ratio of 10:1.
  f. Manually mix Curing Agent and Base Agent in the plastic cup using a plastic knife.
    ***Note:*** Do not mix too fast or the solution will spill. Make sure the entire solution is well-mixed and homogenous. The solution will have gone from transparent to somewhat opaque, with many small bubbles present.
  g. Put the square plastic dish with no lid on the balance and tare.
  h. Carefully pour 7 g mixed solution in the square dish. Use the knife to scrape or stop the flow as needed.
    ***Note:*** Try to pour 7 ± 0.25 g solution; the maximum error should be less than 0.5 g. This creates a sheet that is 1 mm in thickness in this size of square dish.
  i. Carefully remove the square plastic dish with mixed solution from the balance. Cover the dish with the lid and place the dish on a flat part of the lab bench at room temperature (22°C–27°C). Leave the mixed solution to cure for a minimum of 24 h.
    ***Note:*** 24 h should be sufficient for curing at room temperature (22°C–27°C). It may take around 36–48 h if the ambient conditions are humid or cool (<22°C).
    ***Alternatives:*** For faster preparation of the PDMS sheet, place the dish with mixed solution in vacuum desiccator for 30 min to degas until the solution is transparent. Then transfer the dish into a 65°C oven to cure for 2–3 h. Make sure the dish is flat and leveled, or the PDMS sheet will be warped.
    ***Note:*** The recipe in Table 2 generates 1 PDMS sheet (7 g) accounting for some loss of material during the mixing process. The solution begins to cure upon mixing and excess cannot be stored for later use.

**Figure 1:**
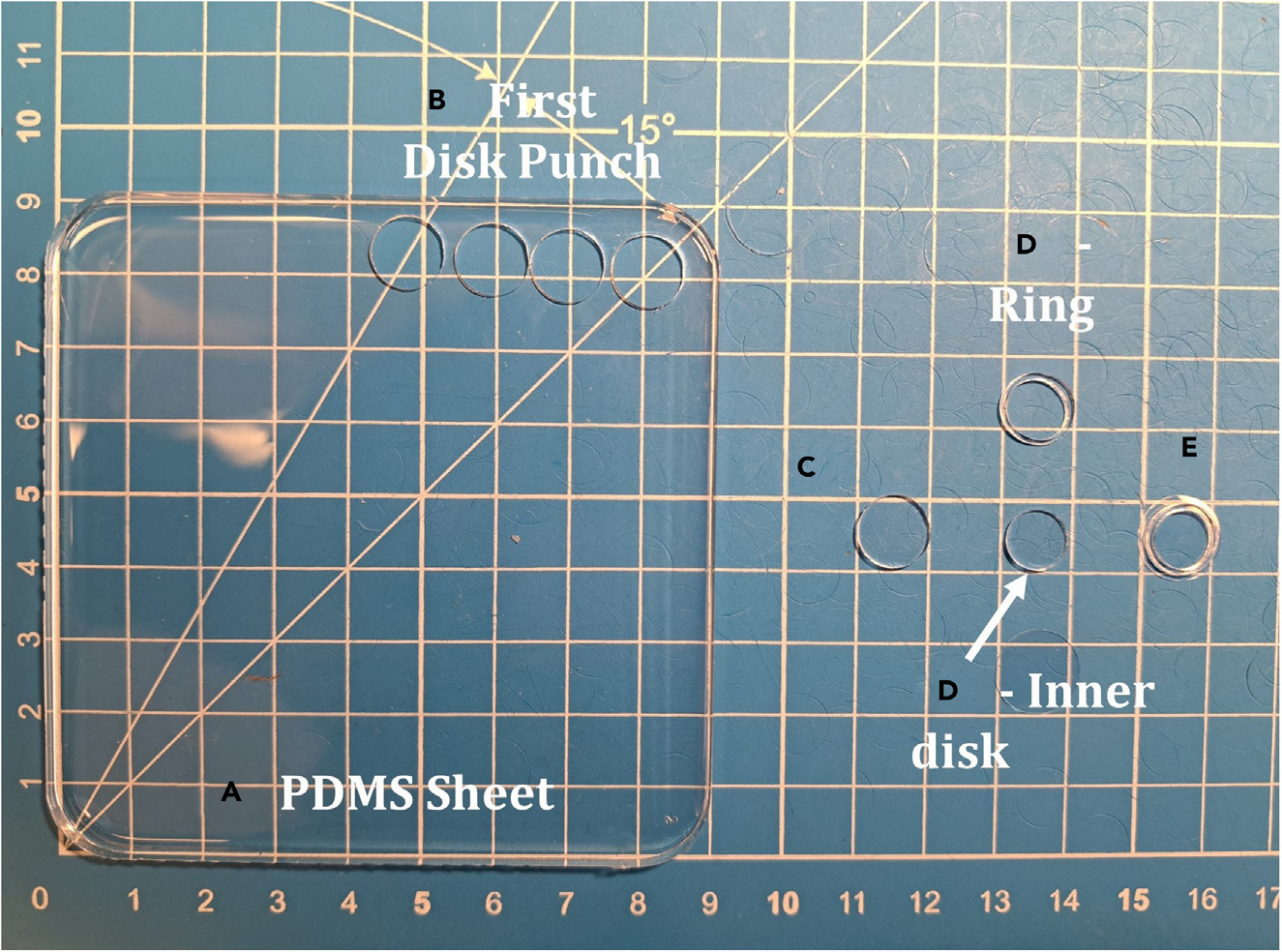
Fabrication of PDMS rings for direct co-culture assays (A) PDMS sheet removed from square dish. (B) Holes remaining from where disks of diameter 10 mm were punched from the top of the PDMS sheet. (C) Initial disk formed after punching and removing from PDMS sheet. (D) Ring (top) created by punching and removing inner disk (lower) from disk shown in (C). Arrow added for clarification. (E) Ring from D placed on glass coverslip, to complete the direct co-culture assay fabrication. Note: All products are shown on a blue, self-healing craft mat with the white squares marking 1 cm by 1 cm areas.
  c. Punch the PDMS sheet with the larger core punch (3/8in, 10-0A, VWR). Press straight down at a 90° angle onto the PDMS sheet.
    ***Note:*** When you are punching the PDMS sheet, you can feel as the punch goes through the PDMS sheet. You can rotate the punch slightly to make sure the sheet is fully cut through by the punch. Approximately 40 such “disks” can be cut from each PDMS sheet.
  d. Line up the disks cut from the PDMS sheet on your flat surface to give yourself more space to punch the smaller hole in each ring.
  e. Carefully press straight down at a 90° angle on the disk by using the smaller core punch (5/16in, 8-0, VWR) to create a PDMS ring.
    ***Note:*** While punching the PDMS sheet, you can feel that the punch goes through the disk. The punch can be rotated slightly to cut the smaller disk from the larger disk fully. Remove the ring from the outside of the core punch using tweezers.
    ***Note:*** To create even thickness and symmetric rings, stay as close as possible to the center of disks when punching the smaller disks. If rings are non-symmetric, they may break and not maintain gel geometry during cell culture.
  f. Mount the PDMS rings on glass coverslips after punching the rings.
    ***Note:*** The coverslips do not need special preparation or cleaning prior to ring mounting. The hydrophobic interactions between the PDMS and the coverslip are sufficient to hold the ECM-based matrices during cell culture experiments.
    ***Note:*** Carefully transfer the rings onto the surface of the glass coverslips using tweezers. You can use either closed tweezers or a gloved-finger to “pat down” the ring to make it adhere to the coverslip. Transfer the finished rings to the empty pipette tip box with an empty rack or other flat, autoclavable container. Make sure the rings are not stacked directly on top of each other. Troubleshooting: problem 1 and problem 2.
  g. Autoclave the rings before transferring into the hood for the vasculogenic ring assay.
    ***Note:*** Let rings cool 24 h before using for cell culture experiments.

6. Fabricating PDMS rings
  ***Note:*** Before punching PDMS rings, make sure that the PDMS sheet is fully cured by touching the edge of the sheet lightly with tweezers. A fully cured sheet will resist deformation and will not be sticky. If the tweezers stick to the surface, continue to cure the sheet at room temperature (22°C–27°C) for another 8–16 h. Alternatively, the dish may be moved to a 65°C oven for faster curing (∼1–2 h).
  a. Insert the tweezers under one corner of the PDMS sheet in the square dish and gently lift the sheet upward.
    ***Note:*** As the sheet is lifted away from the dish, the user’s non-dominant hand can further support the sheet. Using the non-dominant hand to lift and support the sheet frees the dominant hand to continue the removal process using the tweezers. The two-handed approach prevents the sheet from falling over onto itself, which can lead to sticking and ripping.
  b. Carefully transfer the PDMS sheet onto a flat working surface and check for irregularities such as bubbles, thickness differences, etc. See Figure 1 for more details.
    ***Note:*** Users will want a cutting board or craft mat to help ensure consistent ring fabrication.

## Key resources table

**Table:** 

REAGENT or RESOURCE	SOURCE	IDENTIFIER
**Antibodies**		

CD31 Monoclonal Antibody (JC/70A) (Concentration 1:200)	Invitrogen	Cat# MA5-13188
CD36 Antibody (Concentration 1:100)	Thermo Fisher	Cat# MA5-32433
ALDH1A1 Antibody (Concentration 1:400)	CST	Cat# 36671S
CD44 Antibody (Concentration 1:1000)	Abcam	Cat# ab9524
FITC Goat Polyclonal Ab to GFP (Concentration 1:500)	Abcam	Cat# ab6662
Goat-anti-Mouse Alexa-Fluor 488 (Concentration 1:500)	Thermo Fisher	Cat# A11029
Goat-anti-Rabbit Alexa-Fluor 488 (Concentration 1:500)	Thermo Fisher	Cat# A11034
Goat-anti-Mouse Alexa-Fluor 647 (Concentration 1:500)	Thermo Fisher	Cat# A21247
Goat-anti-Rabbit Alexa-Fluor 647 (Concentration 1:500)	Thermo Fisher	Cat# A21245

**Chemicals, peptides, and recombinant proteins**		

DOWSIL™ Sylgard 184 Silicone Elastomer Curing Agent ˆ	Fisher Scientific	Cat# NC0162601
DOWSIL™ Sylgard 184 Silicone Elastomer Baseˆ	Fisher Scientific	Cat# NC0162601
Albumin bovine fraction V(BSA)	RPI Research Products	Cat# A30075-500.0
10% formalin	Fisher Scientific	Cat# SF100-4
Tween-20	RPI Research Products	RPI Research Products
DMEM, 1×	Corning	Cat# 10-013-CV
DMEM (1×, high glucose)	Gibco	Cat# 11995-065
DMEM/F12(1:1) (1×)	Gibco	Cat# 11039021
FBS, 1×	Gibco	Cat# 10437-028
EBM-2 Basal Medium	Lonza	Cat # CC-3156
EGM-2 SingleQuotsˆ	Lonza	Cat# CC-4146
L-glutamine	Thermo Fisher	Cat# 25030149
EGF	PeproTech	Cat# AF-100-15
Hydrocortisone	Sigma-Aldrich	Cat# H0888
Non-essential amino acids	Thermo Fisher	Cat# 11140-050
Sodium pyruvate	Thermo Fisher	Cat# 11360070
Penicillin-Streptomycin Solution	HyClone	Cat# SV30010
Penicillin/Streptomycin 10,000×	Thermo Fisher	Cat# 15140-122
PBS	Corning	Cat# 21-040-CV
DPBS(1×)	Gibco	Cat # 14190-144
0.05% Trypsin	Corning	Cat# 25-052-CV
0.25% Trypsin	Gibco	Cat# 25-052-CV
Fibrinogen from bovine plasma	Sigma	Cat# F8630
Thrombin from bovine plasma	Sigma	Cat# T4648-1KU
SYBR Green Master Mix	Applied Biosystems	Cat# 4367659
BrightGreen 2× qPCR MasterMix	Coderegenesis	Cat# MasterMix-ES

**Critical commercial assays**		

RNeasy Mini Kit	Qiagen	Cat# 74104
High-Capacity cDNA reverse transcriptaseˆ (Includes RT Buffer, dNTP Mix, TR Random Primers, and Reverse Transcriptase)	Thermo Fisher	Cat# 4368813

**Deposited data**		

Data from Indirect co-culture assay for stem markers	Guo et al.^2^	N/A

**Experimental models: Cell lines**		

BON-1 (BON)	Gift from Dr. Hellmich, University of Texas, Galveston	N/A
MCF-7	ATCC	Cat# HTB-22
HMVECi-D	Best et al.^3^	N/A
Human microvascular endothelial cells (HMEC-1 or HMEC)	ATCC	Cat# CRL-3243
Cancer-associated fibroblast (CAF)	Alspach et al.^4^	N/A

**Oligonucleotides**		

CD31 Primer	NCBI Primer Bank	Table 1
CD24 Primer	Wang et al.^5^	Table 1
CD36 Primer	NCBI Primer Bank	Table 1
CD44 Primer	NCBI Primer Bank	Table 1
MYC	NCBI Primer Bank	Table 1
KLF4	IDT Bank	Table 1
ALDH1A1	NCBI Primer Bank	Table 1
PRKD-1	Sequence from IDT	Table 1

**Software and algorithms**		

Real Statistics Resource for Excel	https://www.real-statistics.com/free-download/real-statistics-resource-pack/	Release 7.6
GraphPad	https://www.graphpad.com/	Version 9
FIJI	Schneider et al.^6^	Release 2.9.0; https://imagej.net/software/fiji/downloads
CellSens Dimensions Software∗	https://www.olympus-lifescience.com/en/software/cellsens/	Version 3.1
AngioTool	https://ccrod.cancer.gov/confluence/display/ROB2/Home	Version 0.5
Just Another Colocalization Plugin (JACoP)	https://imagej.net/plugins/jacop	Version 2.1.1
Extended Depth of Field Plugin	https://imagej.net/plugins/extended-depth-of-field	Release 2.0.1

**Other**		

6-well plate∗	Falcon	Cat# 353502
0.4 μm transparent PET membrane	Falcon	Cat# 353090
Tissue culture plate 24 wells, flat bottom∗	Fisherbrand	Cat# FB012929
Round class coverslip circle	Fisher Scientific	Cat# 22-293-232P
Square Petri dish∗	Fisher Scientific	Cat# 50-190-0277
Clear plastic cup	Fisher Scientific	Cat# NC1480232
Tweezers∗	Fisher Scientific	Cat# S95308
Plastic knives∗	Fisher Scientific	Cat# NC1248513
Empty pipette tip boxes ∗	Fisher Scientific	Cat# 02-707-418
Craft mat∗	Amazon	B01LAAHUJ2
Hollow steel core punch set ∗ˆ	VWR	Cat#30002-292
Low-retention syringe filter	Fisher Scientific	Cat# SLGPR33RS
Centrifuge tubes (500 μL)∗	Fisher Scientific	Cat# 07-200-186
Centrifuge tubes (1.5 mL)∗	Fisher Scientific	Cat# 05-408-137
Conical tubes (15 mL)∗	Fisher Scientific	Cat# 14-959-53A
Tabletop centrifuge for cell culture∗: Eppendorf 5810R	Fisher Scientific	Cat# 05-413-112
Vacuum desiccator∗	Fisher Scientific	Cat# 08-594-15C
Oven∗	Fisher Scientific	Cat# 15-103-0508
Inverted epifluorescence microscope∗ : Olympus IX-83	Olympus	Cat# IX-83

***Note:*** Items marked with ˆ are sold as kits. Items marked with ∗ may be substituted depending on users’ preferences and availability.

## Materials and equipment

***Note:*** For the indirect co-culture, the authors recommend the use of specific 6-well plates and transparent PET membranes (See key resources table for vendor and catalog numbers). If the recommended materials are not available, users should verify that the thickness of the well plate and height of the membrane system are compatible and will not touch during culture.

***Note:*** For these studies, HMEC-1 and MCF7 cell lines were purchased from the ATCC. The BON-1 cell line was generously provided by Dr. Mark Hellmich (University of Texas, Galveston, TX). The CAF cell line was previously generated by immortalizing a breast cancer patient specimen and is previously described.^4^

**Table:** MCF-7 cell culture medium

Reagent	Final concentration	Amount
DMEM	N/A	450 mL
FBS	10%	50 mL
100× Penicillin/Streptomycin	1×	5 mL

**Table:** BON cell culture medium

Reagent	Final concentration	Amount
DMEM/F12	N/A	450 mL
FBS	10%	50 mL
100× Penicillin/Streptomycin	1×	5 mL

**Table:** HMVECi-D culture medium

Reagent	Final concentration	Amount
EBM-2 Basal Medium	N/A	500 mL
EGM-2 SingleQuots	N/A	1 kit

**Table:** HMEC culture medium

Reagent	Final concentration	Amount
MCDB 131 Medium	N/A	425 mL
EGF	10 ng/mL	10 μL
Hydrocortisone	1 mg/mL	2 mL
FBS	10%	50 mL
L-glutamine	5%	25 mL

**Table:** CAF culture medium

Reagent	Final concentration	Amount
DMEM	N/A	450 mL
FBS	10%	50 mL
L-glutamine	1%	5 mL
Non-essential Amino Acids	1%	5 mL
Sodium Pyruvate	1%	5 mL
100× Penicillin/Streptomycin	1×	5 mL

***Note:*** All media are stable for 1 mo at 4°C.

Materials and Supplies:

• Square plastic dish: to cure, maintain the PDMS sheet.
• Clear plastic cup: to contain the PDMS mixed solution.
• Plastic knife: to mix the PDMS solution.
• Self-healing craft mat: to cut and move PDMS disks and rings.
• Core punches: to punch the holes/rings.
• Forceps/tweezers: to place the ring in 24-well plate for moving rings and coverslips.
• Round glass coverslip circles: to place the PDMS ring on.
• Empty pipette tip box (with rack): to put the PDMS rings in and autoclave before transferring to cell culture hood, for storing and autoclaving rings.
• Low retention filters: to sterile filter fibrinogen solution prior to cell culture steps.
• Eppendorf centrifuge 5810R: to cells and fibrinogen solution.
• Vacuum desiccator: to remove of the bubbles in PDMS sheets (optional).
• 65°C oven incubator: to speed PDMS curing time (optional).

Other Solutions:

• Fibrinogen Solution: add 20 mg fibrinogen to 1 mL DPBS.

The above recipe generates enough fibrinogen for 24 ring samples. Solution can be scaled up as needed depending upon the number of samples. Information on solution storage and stability is found in the table below.

***Note:*** Depending on volume needed for experiments, this can be done in either a 1.5 mL tube or a 15 mL conical tube. The tube should be incubated at 37°C in an incubator or water bath to fully dissolve the fibrinogen. When possible, this solution should be made fresh on the day of seeding cells in the direct co-culture assay.

• Thrombin Solution: add 10 mg BSA to 10 mL DPBS; dilute stock thrombin powder to 50U/mL in this solution.

Thrombin concentration depends on active units (U) per mg and varies based on lot number/manufacturer. This information is usually located on the product label. Thrombin solutions do not maintain stability in freeze/thaw cycles and should be aliquoted in 50–100 μL batches for experimental purposes. Information on solution storage and stability is found in the table below.

• Blocking Solution: add 2 g BSA and 0.1 mL Tween-20 to 100 mL DPBS.

Information on solution storage and stability is found in the table below.

• EGF Solution (For HMEC medium): add 0.5 mg EGF to 5 mL sterile filtered water; aliquot into 50–100 μL units.

Information on solution storage and stability is found in the table below.

• Hydrocortisone Solution (For HMEC medium): add 25 g to 10 mL sterile filtered water.

Information on solution storage and stability is found in the table below.

**Table:** Solution Stability and Storage Temps for Reagents and Solutions

Reagent or solution	Storage temperature	Stability time
Fibrinogen Solution (20 mg/mL)	37°C (Water Bath)	Up to 12 h
Thrombin Solution (50U/mL)	−20°C	Up to 12 mo
Media Formulations (See above)	4°C	Up to 1 mo
EGF Solution (0.1 mg/mL)	−80°C	Up to 12 mo
Hydrocortisone Solution (0.25 g/mL)	−20°C	Up to 12 mo
Blocking Solution	4°C	Up to 12 mo

***Alternatives:*** Please see below for alternatives for some materials or equipment.

• Fibrinogen: Users may choose to add additional extracellular matrix components, such as collagen, to the fibrinogen solution based on their studies of interest.
• Minor supplies: Items such as tweezers, culture dishes, and consumable plastics may be swapped for alternative, similar items based on user preference and/or availability from manufacturers. These items are marked with an asterisk (∗) in the KRT.
• Equipment: Items such as the table top centrifuge, vacuum desiccator, and inverted microscope may be swapped for similar products based on availability in users’ labs. These items are marked with an asterisk (∗) in the KRT.

## Step-by-step method details

### Indirect co-culture

**Timing: 4 days**

This protocol is an example of the indirect co-culture study for the role of vascular ECs in cancer cell stemness. Towards this end, the cancer cells are seeded in the 6-well plate while the ECs are plated in the transwell. Seed the cells that you need to characterize in the 6-well plate.

1. Day 1: Pre-warm the media PBS, and trypsin in a 37°C water bath. Clean the cell culture hood and turn on UV light for 30 min before the experiment starts.
2. Check the cell status and make sure that the confluences reach about 80%–90%.
3. Remove media, wash the cells using warm PBS once, and detach the cells from 10 cm dish by incubating with trypsin for 2 min.
4. Add medium to trypsin in 2:1 ratio (medium: trypsin) to neutralize the trypsin.
5. Centrifuge cells at 200–300 × *g* for 5 min at room temperature (22°C–27°C) to pellet cells.
6. Discard the medium and re-suspend the cells with 2 mL medium for cell counting.
7. Seed 2 mL cancer cells with a density of 3.0 × 10^5^ cells/mL in each well of a 6-well plate. The total cell number in each well is 6.0 × 10^5^ cells. This well plate contains the cancer cells that will be tested for stem markers in later steps.
8. Prepare a second 6-well plate by carefully putting the transwell inserts in the 6-well plate. This is for loading the control or experimental cells onto the insert prior to starting indirect co-culture.

**Figure 2:**
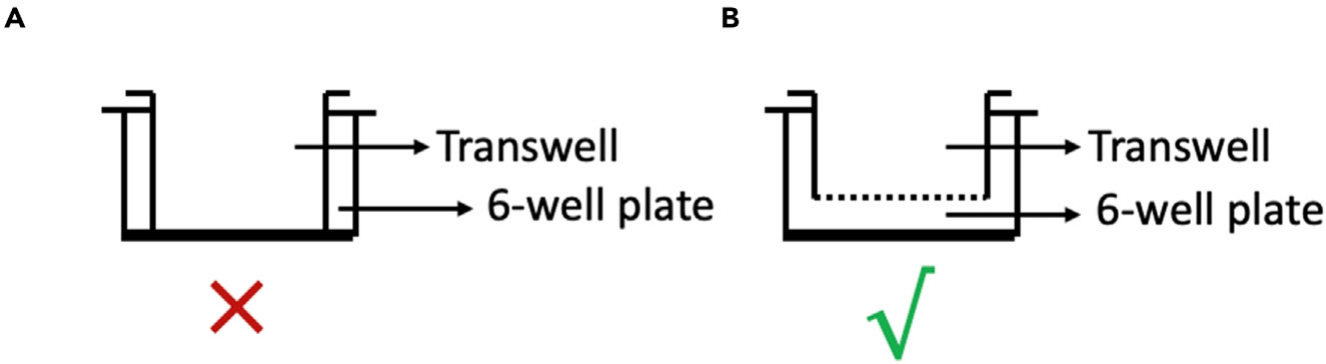
Proper configuration for indirect co-culture studies (A) Inappropriate fitting of the transwell insert due to contact with the well plate. (B) Appropriate configuration of insert into 6-well plate for indirect co-culture assay.

**CRITICAL:** Check if the insert can match up with the 6-well plate before starting the experiment. The insert cannot just “stand” in the well. There must be some gap between the insert and the well of the 6-well plate (Figure 2).

**Figure 3:**
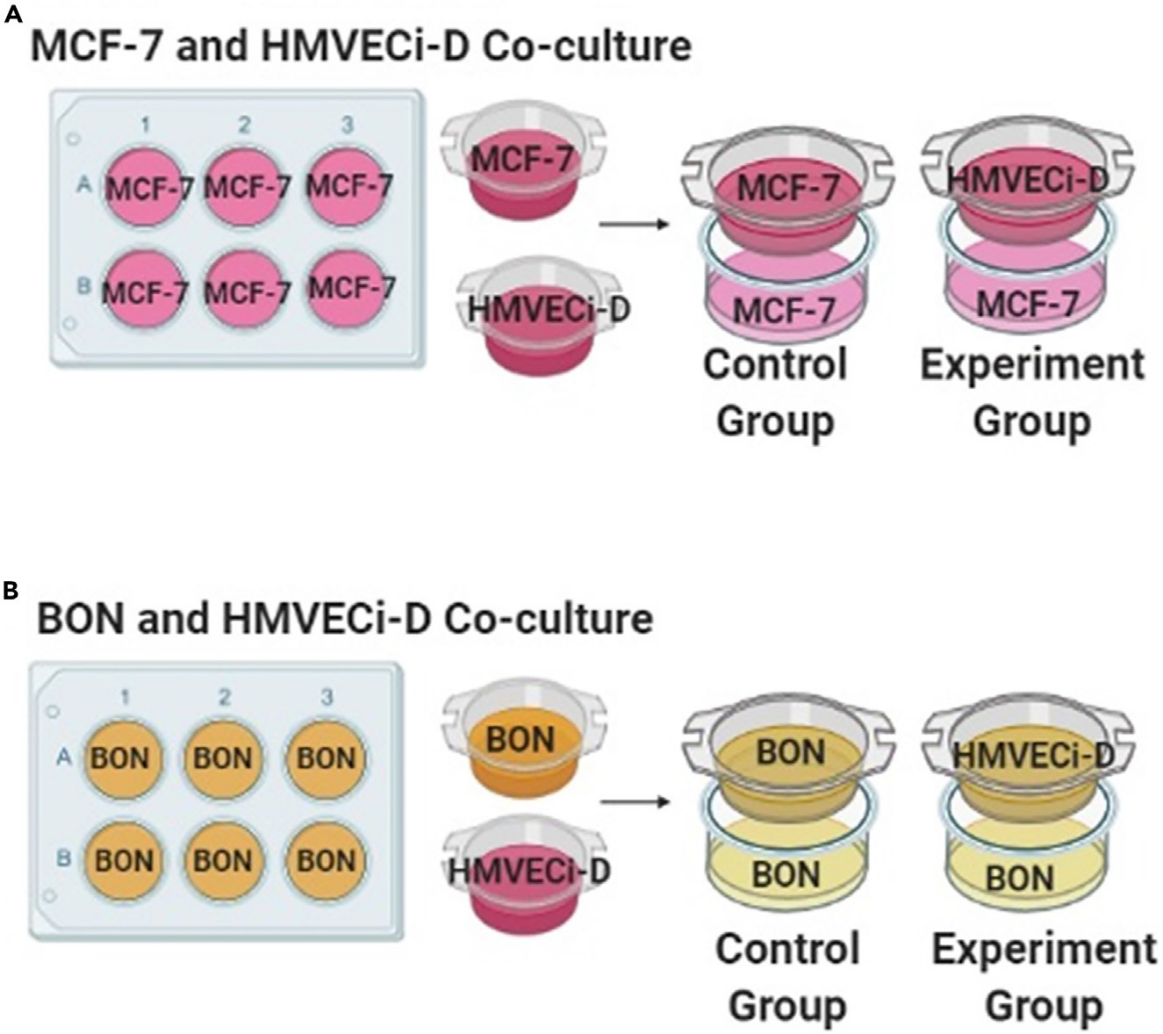
Seeding protocols for indirect co-culture studies (A and B) (A) MCF-7 and (B) BON cells indirect co-culture with HMVECi-D cells schematic. Cancer cells are seeded in a 6-well plate for 24 h (Day 1) while both cancer cells and HMVECi-D cells are cultured in inserts in a second well plate for 24 h (Day1). After 24 h culture, media is changed and the inserts are added to 6-well plates containing only cancer cells (Day 2). Inserts seeded with cancer cells are controls. For seeding concentrations/densities, please see Table 3.

***Note:*** Because cells need some time to attach to the bottom of transwells, seed the control and experimental groups in the transwells 24 h before co-culture begins.

***Note:*** In the control group, cancer cells should be cultured onto the insert.

***Note:*** In the cancer microenvironment, the cancer cell number is much higher than the number of vascular ECs. Therefore, we designed two ratios between the cells in the insert (ECs or cancer cells) and the cell seeded in 6-well plate (cancer cells). The ratio is defined as which are cells in insert to cells in 6-well plate ratio and the following ratios were tested 1:5 or 1:10.

***Note:*** The EGM-2 medium is used for HMVECi-D culture.

9. Seed cells onto the transwell inserts. The experimental group is HMVECi-D, which tests how the ECs alter cancer stem markers. Use BON or MCF-7 cells as control groups in the inserts. Culture at 37°C and 5% CO_2_ for 24 h before starting the co-culture (Table 3; Figure 3).

**Table 3:** Seeding densities for indirect co-culture studies used in this protocol

Ratio	Control group (cancer) or experimental group (HMVECi-D)
1:5	120,000 cells/well
1: 10	60,000 cells/well

10. Day 2: Aspirate the medium in the 6-well plate and transwell insert.
11. Replace the endothelial cell medium with cancer cell medium.

***Note:*** Since the two cells are co-cultured, the medium should be the same. The minimum recommended volume for the lower chamber is 2 mL, with a maximum of 2.6 mL suggested. The minimum volume for the insert is 1 mL, with a maximum of 1.5 mL.

12. Put the insert into the corresponding wells in the 6-well plate.
13. Day 4: After 48 h co-culture, wash the cancer cells in the 6-well plate twice with PBS and lyse to collect total RNA using the instructions on the RNeasy Mini Kit Protocol.

***Note:*** The manufacturer’s online protocol may be found at the following link: https://www.qiagen.com/us/products/discovery-and-translational-research/dna-rna-purification/rna-purification/total-rna/rneasy-kits?catno=74104.

14. Quantify the RNA concentration by Nanodrop and use molecular biology-grade water to dilute samples as needed to maintain equal concentrations.
15. Prepare the mixture for reverse transcription in PCR tubes following the recipe in Table 4.

**Table 4:** cDNA recipe for reverse transcription protocol

Reagents	Final concentration	Volume
Total RNA	1,000–2,000 ng	10 μL
RT Buffer∗	1×	2.0 μL
dNTP Mix∗ (100 mM)	1×	0.8 μL
RT Random Primer∗	1×	2.0 μL
MultiScribe™ Reverse Transcriptase∗	N/A	1.0 μL
Nuclease-free H_2_O	N/A	4.2 μL
**Total**	**N/A**	**20** μL

***Note:*** Items marked with ∗ are included in the High-Capacity cDNA reverse transcriptase kit described in the KRT.

***Note:*** The amount of RNA that can be reverse transcribed is 2,000 ng based on the optimized protocol. Use up to 2 μg of total RNA per 20 μL reaction. Solution volumes may be scaled as necessary to ensure sufficient total volume for the reaction.

16. Perform RNA reverse transcription following the protocol outlined in Table 5.

**Table 5:** Thermal cycles for RNA reverse transcription

Steps	Temperature	Time
Annealing	25°C	10 min
DNA Polymerization	37°C	120 min
Enzyme Deactivatoin	85°C	5 min
Hold	4°C	Forever

**Pause point:** The cDNA is stable at −20°C for 2 weeks; for longer storage times, −70°C storage should be utilized.

17. Prepare master mix for RT-qPCR reaction in PCR tubes based on Table 6.

**Table 6:** Master mix for RT-qPCR reaction

Reagents	Final concentration	Volume (μL)
Bright Green 2× qPCR MasterMix	1×	10
Forward Primer (10 μM)	300 nM	0.6
Reverse Primer (10 μM)	300 nM	0.6
cDNA	Variable	Variable
Nuclease-free Water	Variable	Variable
**Total**	**N/A**	**20**

***Note:*** Make sure the primer concentration for RT-qPCR is 10 μM. The concentration of the primer from IDT is 100 μM. If users are limited in reagents, a 10 μL total volume basis could also be used for the qPCR system. Reduce the volume of each reagent in Table 6 by a factor of 2.

18. Run RT-qPCR following the thermocycler protocol shown in Table 7.

**Table 7:** Thermocycler RT-qPCR protocol used in this study

Steps	Temperature	Time	Cycles
Enzyme Activation	95°C	10 min	1
Denaturation	95°C	15 s	40
Annealing/Extension	60°C	60 s	1
Hold	4°C	Forever

### Direct co-culture or vasculogenic ring assay

**Timing: 7 days**

This assay generates a small disk representing a 3D microtissue that models the tumor microenvironment; each sample can contain multiple cell types to permit investigations of both biochemical interactions between cells as a result of secreted factors as well as biomechanical interactions through cell-cell adhesions or matrix remodeling. The benefit of using a PDMS ring on a glass cover slip to hold the ECM-based gels, as opposed to using glass-bottom well plates, is that this configuration provides a reliable and consistent geometry between studies. The sides of the PDMS ring create a barrier that forms the fibrin gel into a regular cylinder, rather than the hemi-spherical shape when cell-gel mixtures are placed directly on a substrate. The glass coverslip also provides a way the entire sample can be removed from the 24 well plate after culture, permitting enhanced flexibility for how samples are processed and imaged. In other words, the technique can be expanded to permit protocols such as embedding in paraffin or OCT for sectioning and IHC processing similar to *in vivo* or clinical specimens to compare *in vitro* microtissue models to these other models.

19. Prepare fibrinogen at a concentration of 20 mg/mL by diluting fibrinogen powder in DPBS without Ca^2+^ or Mg^2+^.

***Note:*** Each ring needs 50 μL fibrinogen; this volume is sufficient to fill the PDMS ring, creating a cylindrical disk. Calculate the amount of fibrinogen that will be needed, based on total rings to be prepared. Include additional reactions in the calculations because 50% of volume can be lost during filtration.

***Note:*** Dilute fibrinogen powder with DPBS outside the hood. Let the tube containing fibrinogen incubate in 37°C water bath for at least 1 h before next steps. Spin the tube at 8–10 × *g* for 2 min at room temperature (22°C–27°C). Sterile filter the fibrinogen by using syringe and 0.22 μm low-retention filter (Millex-GP SLGPR33RS) (See key resources table) in cell culture hood. Put the fibrinogen back into the 37°C water bath to keep warm while harvesting cells.

20. Pre-warm endothelial cell medium, BON cell medium, CAF medium, trypsin, PBS, and DPBS.
21. Spray 70% ethanol to clean the hood and turn on UV light to sterilize the entire cell culture hood for at least 30 min.
22. Transfer PDMS rings on coverslips into each well of a 24-well plate in the tissue culture hood.

***Note:*** 24-well plates should be used in the experiment since the size of PDMS rings are the same as the wells in 24-well plate.

**CRITICAL:** Since the PDMS rings attached on the glass coverslips are transparent, make sure that the PDMS rings face upward. Check each PDMS rings by touching them with a tweezer. If the PDMS ring is facing upward correctly, you should feel the soft PDMS ring instead of the rigid glass. The PDMS may provide a slight “bounce” feel when the tweezers are pushed into it. The rings should remain on the coverslips for the duration of the experiment.

23. Harvest the cells by detaching and collecting all the cells in a 50 mL tube and take 10 μL for cell counting.

***Note:*** 0.5% trypsin can be used to detach the cells from a 10 cm dish.

***Note:*** Find the appropriate cell number in each ring. If too many cells are used, the cells in the rings will be crowded and could cause gel collapse; if the cell density is not high enough, the ECs and BON cells cannot proliferate in the ring. We tested different cell numbers in several pilot experiments. The maximum recommended number of cells per ring is 1 × 10^6^. For more information on cell density optimization, please see the limitations section below.

***Note:*** We tested two different conditions: (1) More ECs than cancer cells (5:5:1 and 10:10:1); (2) More cancer cells than ECs (1:5:5 and 1:10:10). These ratios are HMEC-1:CAF:BON cells. We also tested 1:5, 1:10, 5:1, and 10:1 HMEC:BON ratios.

24. Resuspend BON, HMEC-1, and CAF cells at appropriate densities following Table 8.

**Table 8:** The BON, HMEC-1, and CAF cell concentrations for the ring assay

Ratio	HMEC-1 total	CAF total	BON total	HMEC-1 concentration	CAF concentration	BON concentration
**Endothelial cell number higher than cancer cells**						

5:5:1	1.84 × 10^5^	1.84 × 10^5^	3.67 × 10^4^	8.37 × 10^6^	8.37 × 10^6^	1.67 × 10^6^
10:10:1	1.93 × 10^5^	1.93 × 10^5^	1.93 × 10^4^	8.77 × 10^6^	8.77 × 10^6^	8.77 × 10^5^

**Cancer cell number higher than endothelial cells**						

1:1:5	5.78 × 10^4^	5.78 × 10^4^	2.88 × 10^5^	2.63 × 10^6^	2.63 × 10^6^	1.31 × 10^7^
1:1:10	3.37 × 10^4^	3.37 × 10^4^	3.37 × 10^5^	1.53 × 10^6^	1.53 × 10^6^	1.53 × 10^7^

***Note:*** Calculate the cell number using the basis of 3 rings for convenience. Each ring needs at least 100K cells, which is at least 300K cells for 3 rings. Three rings need 22 μL of each cell line (7.3 μL for each ring). Three rings need 66 μL of total cell suspension (e.g., - 22 μL for each cell line in a tri-culture experiment).

**CRITICAL:** While only a small number of cells is needed in each ring, the concentration of cells is fairly high. Make sure that the three cell lines are ready before starting the ring assay; all of the BON, HMEC-1, and CAF cell confluence values should be 80%–90% in each 10 cm dish. This should ensure there are sufficient cells to load 15–36 rings, depending on desired final concentration (See Table 8). To get these cell concentrations, count the number of cells harvested, then calculate the volume of medium required to dilute the cells to reach the needed concentration for the ring assay.

**CRITICAL:** HMEC-1 cells cannot form blood vessel-like structure without the support of stromal cells. For these studies we used CAFs.^1^^,^^4^

25. Make cell suspensions in sterile microcentrifuge tubes for loading rings.

***Note:*** It is recommended to use 500 μL tubes, as it is somewhat easier to hold them while pipetting in future steps, although 1.5 mL tubes will work as well.

26. Mix BON, HMEC-1 and CAF cell suspensions (22 μL for each cell type) with 9 μL of thrombin.

***Note:*** Thrombin was solubilized in 0.1% BSA in DPBS at 50U/mL, aliquoted at 50–100 μL per tube, and stored in −20°C freezer. This solution is stable for at least 12 mo when stored properly.

**CRITICAL:** Thrombin aliquots do not survive multiple freeze/thaw cycles.

**CRITICAL:** Mix the BON, HMEC-1, and CAFs first, then add thrombin and mix thoroughly. The final volume should be 75 μL.

27. Add 75 μL of fibrinogen into the cell suspension, mix three times using the pipette via trituration, then turn the pipette to 50 μL quickly and add 50 μL to each ring, careful to not introduce bubbles.

**CRITICAL:** Store the fibrinogen in a 37°C water bath until starting this step.

**CRITICAL:** Fibrinogen quickly polymerizes into fibrin upon addition of thrombin. Finish this step as quickly as possible. Use a 100 μL pipette and change the volume from 75 μL to 50 μL quickly. Do not change pipette tips.

***Note:*** To prevent bubbles, avoid pressing the pipette all the way down. Troubleshooting: problem 2 and problem 3.

***Optional:*** If users are having difficulties with changing pipette volume quickly, prepare two pipettes: one set to 75 μL, another set to 50 μL. Change the pipette immediately after you mix fibrinogen three times by 75 μL pipette.

28. With the dispensing button still depressed, swirl pipette tip around to fully spread gel mix throughout whole ring.

***Note:*** Due to the three rings needing to be finished together, the time spent on each ring should be as short as possible. We recommend transferring 50 μL to each ring, then going back and swirling the solutions to get even dispersion.

**CRITICAL:** The mixture will begin to polymerize within 30 s.

Users should move quickly to ensure the polymerization does not happen inside the pipette tip.

Finish three rings within 30 s. Troubleshooting: problem 2.

29. Move to incubator and allow gels to set for up to 30 min.

***Note:*** Gels may sit for a maximum of 1 h before media is added; any longer than 1 h and gel stability is compromised, and cells may begin to die.

30. Add 1 mL of EGM-2 medium in the wells with the mixture of BON, HMEC, and CAF cells.

***Note:*** Can use minimum 500 μL medium per well media if necessary.

31. Check the cell status every day and change media every 2 days, for 7 days of total culture time before proceeding to step 32. Troubleshooting: problem 4.

***Note:*** Samples with both HMEC-1 cells and CAFs may begin to generate vasculature structures by day 3 or 4.

***Note:*** When discarding the medium by vacuum aspiration, do not touch the cells or gels in PDMS rings with the tip of aspirating tube. The fibrin gel can be aspirated by vacuum flasks. Touch the wall of each well to remove the medium carefully.

***Note:*** A manual pipette may be used if preferred to remove the media.

### Ring assay fixation and staining

**Timing: 3 days**

This section describes the protocol for fixation and immunofluorescence (IF) staining for cells cultured in the 3D *in vitro* ring assay described above. In addition to IF-based techniques, users should consider utilizing cell lines with fluorescent tags or reporters for proteins of interest in their specific studies. Including pre-labeled markers can simplify and shorten the staining process.

32. Day 1: Remove the 24-well plate with rings from the incubator and transfer to a lab bench for histological analysis.

***Note:*** This is Day 1 of the Staining protocol and Day 8 of the co-culture protocol described in the previous section.

***Note:*** All further steps can be performed in non-sterile conditions.

33. Wash the rings with ∼500 μL PBS twice, each for 5 min.

***Note:*** Use a vacuum aspirator flask on the bench to aspirate the media from the 24-well plate. The aspiration tube tip can only touch the wall of each well to avoid disturbing the cells or gel in the ring.

**CRITICAL:** The vacuum flask should contain 10% bleach.

34. Fix the rings using 10% formalin for 20 min on an orbital shaker at room temperature (22°C–27°C).

***Note:*** Add ∼300 μL of 10% formalin in each well. Remove 10% formalin by pipette and dispose according to hazardous waste protocols.

***Note:*** It is not necessary to quench after fixation for immunofluorescence staining protocols.

35. Wash the rings with PBS on an orbital shaker at room temperature (22°C–27°C) three times, each for 5 min.

**Pause point:** You may stop here by wrapping the 24-well plate in aluminum foil and storing it at 4°C. Samples may be stored for 3–4 weeks before staining.

36. Prepare 2% BSA blocking buffer by dissolving 2 g BSA powder in 100 mL PBS with 0.1% Tween-20.

***Note:*** BSA dissolution in PBS may be sped up by incubating at 37°C for ∼20 min.

37. Remove PBS and add 200 μL blocking buffer to block for 1 h at room temperature (22°C–27°C) on orbital shaker.
38. Aspirate the blocking buffer. Incubate each ring with 200 μL primary antibody solution.

***Note:*** Check the dilution for each antibody to calculate total volume of antibody needed. Dilute antibody in blocking buffer. Wrap the 24-well plate in aluminum foil and store at 4°C fridge overnight (12–24 h) on an orbital shaker.

***Note:*** If using multiple primary antibodies to stain for multiple targets, these can be incubated simultaneously if the host species are different.

39. Day 2: Remove the primary antibody solution and wash the rings with phosphate-buffered saline/tween (PBST) four times, each wash for 20 min at room temperature (22°C–27°C) on the orbital shaker.

***Note:*** PBST was prepared by 1× PBS with 0.1% Tween-20.

40. Add 200 μL per well of secondary antibody diluted in blocking buffer.

**CRITICAL:** Check the secondary antibody dilution and the animal species of primary antibody. Check the fluorophore color to make sure each target can be distinguished.

41. Wrap the 24-well plate in aluminum foil and store at 4°C fridge overnight (12–24 h) on an orbital shaker.
42. Day 3: Remove the secondary antibody and wash the rings with PBST four times, each wash for 20 min at room temperature (22°C–27°C) on the orbital shaker.
43. Stain the cells with DAPI for 20–30 min at room temperature (22°C–27°C) on the orbital shaker.

***Note:*** Check the dilution concentration of DAPI and the solution used for dilution.

44. Discard the DAPI and wash the rings at least once, for a minimum of 5 min, with PBST to remove excess DAPI.
45. Staining is complete and image the rings on an inverted fluorescence microscope.

**Pause point:** You can store the rings at 4°C by wrapping the 24-well plate with aluminum foil. Systems will retain stain for 4–6 weeks under these conditions.

### Ring imaging and analysis

**Timing: 1 day (imaging); variable (analysis)**

This protocol describes an imaging technique for the 3D co-culture models described in the previous sections based on IF staining. Users should optimize imaging parameters based on their specific targets of interest and staining protocols. The analysis techniques described here were used in the generation of data and figures shown in the expected outcomes section. This is a semi-quantitative technique used to provide direct comparison between sample groups generated during the co-culture assay. The length of time needed to complete the analysis is dependent on how many samples users have in an experiment and computational capacity of users’ desktops or laptops.

46. Image rings on inverted epifluorescence microscope.

***Note:*** In this study, an Olympus IX83 system with CellSens Dimensions software (Version 3.1) was used for image collection.

***Note:*** Z-stack images were collected for each ring at 100 μm thickness and 2 μm step size. Exposure times were optimized based on negative controls (no primary antibody incubation). Troubleshooting: problem 5.

47. Project the 3D z-stacks to 2D using FIJI and the Extended Depth of Field plugin for FIJI.^7^

***Note:*** This allows for analysis of a 3D volume using 2D image processing software.

48. For vascular growth analysis, use AngioTool to calculate total vessel growth per image area as well as average vessel length.^8^

***Note:*** Images must first be saved as a false-color JPEG to be compatible with AngioTool software.

49. To ensure that stem markers observed in immunofluorescence studies are associated with BON cells, we used the Just Another Colocalization Plugin (JaCoP) in FIJI to determine the Pearson’s coefficient for specific markers with the BON cells.^9^

## Expected outcomes

### Indirect co-culture assay results

**Figure 4:**
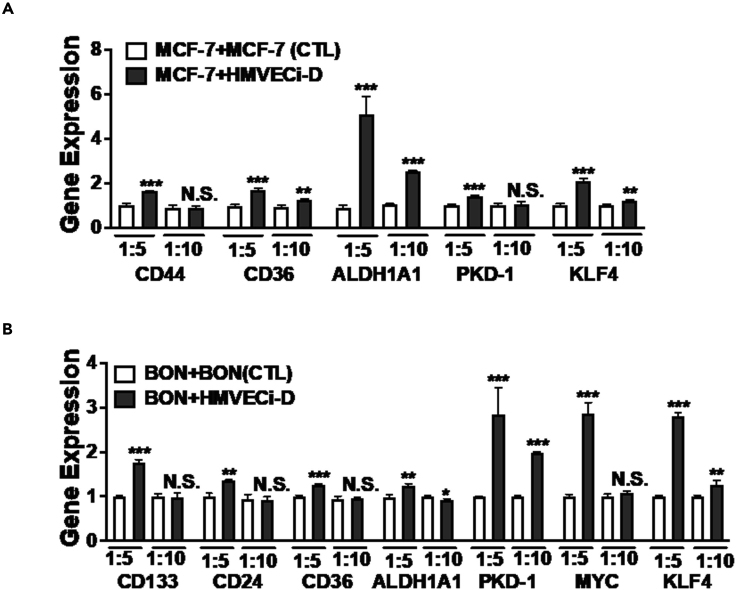
Changes in gene expression in breast cancer cells via indirect interactions of microvascular endothelial cells with breast cancer stem-like cells (A) Co-culture of breast cancer cells and endothelial cells increased expression of breast cancer stem cell (BCSC) markers and stemness-associated genes. Total RNA in MCF-7 cells cultured in a 6-well plate was collected for detection of stemness-associated genes by RT-qPCR. 1:5 ratio of MCF-7 and HMVECi-D increase more stemness than 1:10 ratio. (B) Co-culture of PNET cells and endothelial cells increased expression of CSC markers and stemness-associated genes. RT-qPCR detected the gene levels of stemness-associated genes of PNET cells cultured in 6-well plate. The results are from triplicate experiments. Data shown are averages + SEM. ∗∗∗p < 0.001, ∗∗p < 0.01, or ∗p < 0.05.

Results from RT-qPCR protocol provide information on relative differences in copies of genes in samples compared to control groups. For this study, we have plotted this as normalized gene expression to control samples consisting of cancer cells on an insert. The indirect co-culture system shows small but significant increases in gene regulation of stem cell markers in MCF-7 and BON cells when introduced to secreted factors from HMVEC-iD cells (Figure 4). Increases in CD36 expression were seen in both MCF-7 and BON cells when exposed to HMVEC-iD cells compared to the control cell groups at the 1:5 ratio. The increases in stem cell marker gene expression are higher at the 1:5 ratio versus the 1:10 ratio for nearly all tested conditions, possibly indicating that the secreted factors from the HMVEC-iD are more potent in upregulating these markers when fewer cancer cells are present.

### Direct co-culture assay results

**Figure 5:**
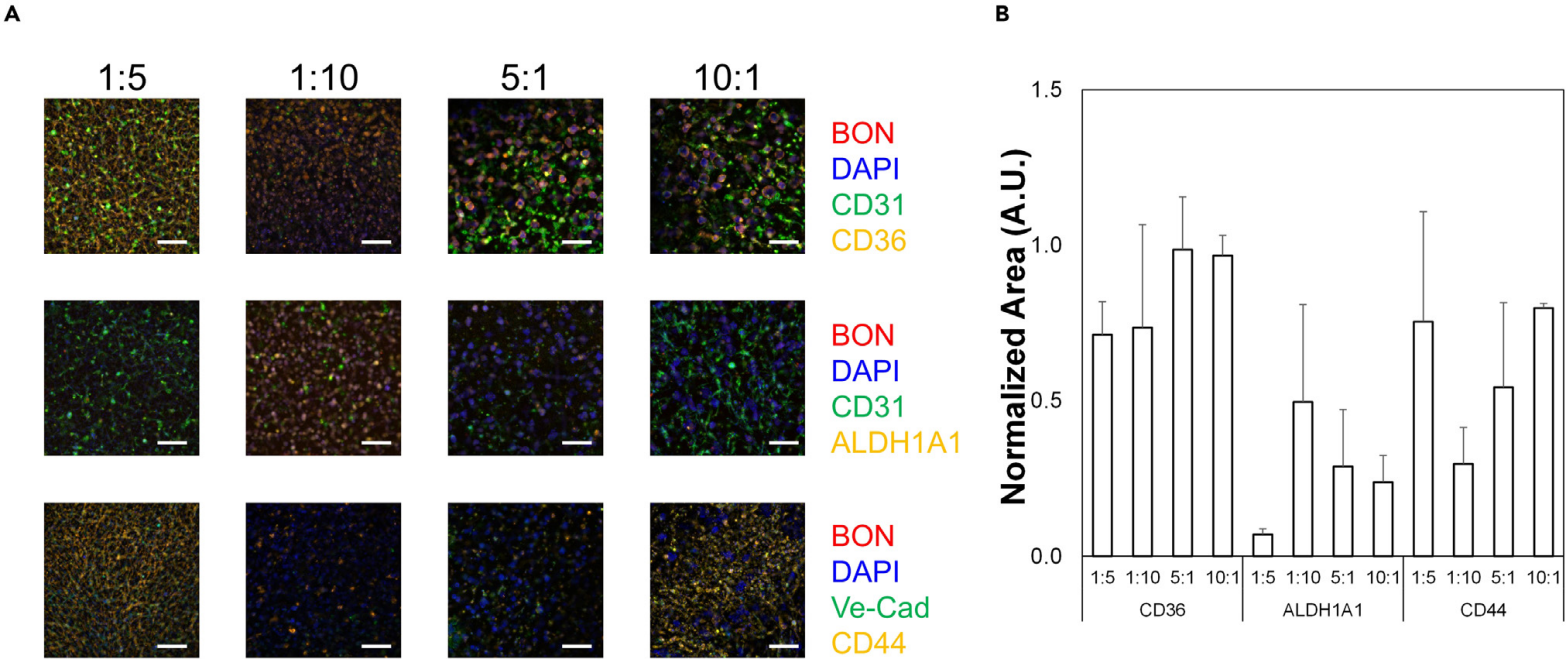
Changes in stem cell markers in BON cells in co-culture with HMEC-1 cells at different ratios (A) Representative immunofluorescence images of 3D ring assays containing differing ratios of HMEC-1 to BON cells. Colors are shown in legends to the right of the images. Scale bars = 250 μm. Images are 100 μm z-stacks projected for maximum focus through the Extended Depth of Field algorithm. These images are then processed with the Analyze Particles measurement algorithm in FIJI to determine total positive area for each stain. (B) Quantification of positive area for each stain relative to DAPI area. The results are from triplicate experiments. No statistical significance was observed for different ratios; however, there are significantly higher levels of CD36 compared to ALDH1A1 for all samples (ANOVA, p < 0.05). Data shown are averages + SEM for triplicate samples. See also Figure S1 and Table S1.

**Figure 6:**
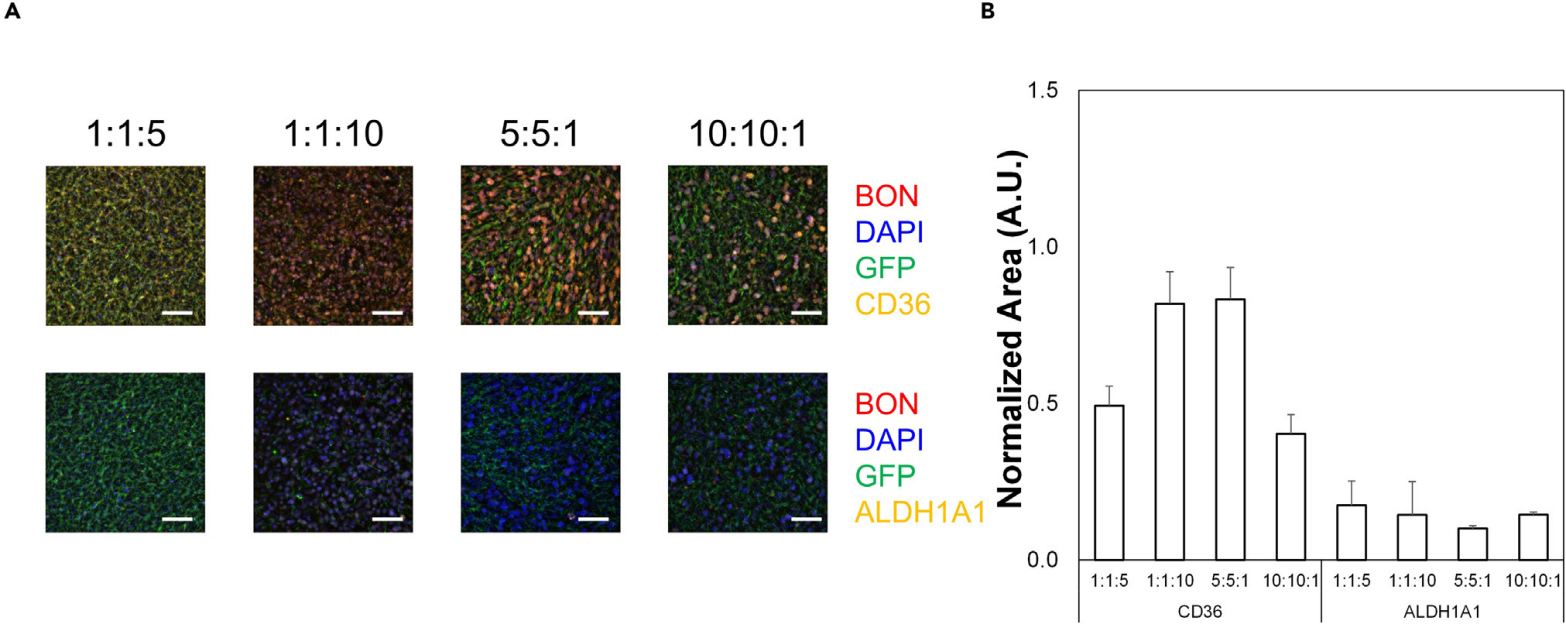
Changes in stem cell markers in BON cells in co-culture with HMEC-1 cells and CAFs at different ratios (A) Representative immunofluorescence images of 3D ring assays containing differing ratios of HMEC-1 to CAFs to BON cells. Colors are shown in legends to the right of the images. Scale bars = 250 μm. Images are 100 μm z-stacks projected for maximum focus through the Extended Depth of Field algorithm. These images are then processed with the Analyze Particles measurement algorithm in FIJI to determine total positive area for each stain. (B) Quantification of positive area for each stain relative to DAPI area. The results are from triplicate experiments. No statistical significance was observed for different ratios; however, there are significantly higher levels of CD36 compared to ALDH1A1 for all samples (ANOVA, p < 0.05). Data shown are averages + SEM for triplicate samples. See also Figure S1 and Table S2.

**Figure 7:**
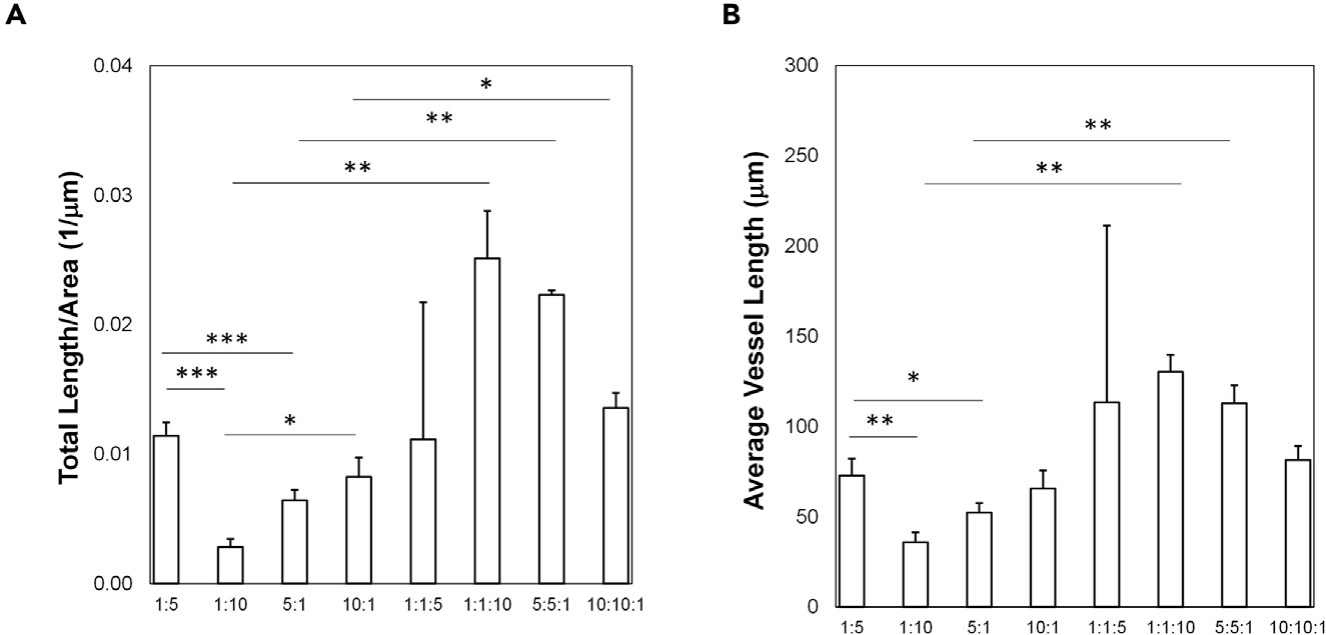
Vascular growth in direct co-culture models (A) Normalized vessel growth (total length per area imaged) shown for different ratios of HMEC-1:BON and HMEC-1:CAF:BON sample groups. (B) Average length of vascular structures in samples with varying ratios of HMEC-1:BON and HMEC-1:CAF:BON cells. The results are from triplicate experiments. The addition of CAFs significantly increased total vascular growth and average length for all ratios tested (ANOVA, p < 0.05). Specific comparisons between groups are shown on the charts. Data shown are averages + SEM for triplicate samples. ∗∗∗p < 0.001, ∗∗p < 0.01, or ∗p < 0.05.

The immunofluorescence images acquired from the direct co-culture assay should show staining for the targets of interest and spatial organization of the multiple cell types (Figures 5A and 6A). Changes in targets can be caused by secreted factors as well as interactions with adjacent cell types not based on soluble ligands. Using FIJI or similar software, users can generate quantitative measurements of the area positively stained for the markers relative to a control (Figures 5B and 6B). In these studies, DAPI was used as a counterstain to normalize expression of all markers to total cell area. The BON cells express tdTomato, while CAFs express GFP, permitting separation of stem cell or endothelial markers between populations. Overall positive area was also calculated for CD31, VE-Cadherin, GFP, or td-Tomato to quantify relative areas of stromal or cancer cell components within the system (Figure S1); no significant differences were observed for any parameter, although increased levels of GFP in all CAF samples may indicate that CAFs proliferated faster than either ECs or BON cells in these samples. The colocalization analysis also described the Pearson’s Correlation Coefficient (PCC), measured via the JaCoP plugin, for the stem markers relative to the BON cells (Tables 9 and 10); our studies demonstrate a strong or very strong correlation for CD36 and CD44 staining with BON cells but not CAFs or HMEC-1 cells. Analysis of PCC for the EC or CAF markers with CD36 and ALDH1A1 showed negligible or weak correlation, indicating that the stem markers are not associated with either of these cell types (Tables S1 and S2). To further highlight the potential of this system, we analyzed CD31 staining in the samples to measure blood vessel growth (Figure 7). Samples with 1:1:10 HMEC-1:CAF:BON demonstrate the highest levels of vascularization of all samples. Therefore, the direct co-culture is useful for monitoring changes in stem cell markers in addition to other characteristics important in the TME, such as vascular growth.

**Table 9:** Pearson’s Correlation Coefficient (PCC) for stem cell markers in HMEC-1:BON cell studies

	Ratio HMEC:BON	PCC for BON:Marker	Interpretation
CD36	1:5	0.731 ± 0.007	Strong
	1:10	0.800 ± 0.071	Strong
	5:1	0.876 ± 0.002	Strong
	10:1	0.795 ± 0.100	Strong
ALDH1A1	1:5	0.195 ± 0.004	Weak
	1:10	0.259 ± 0.060	Weak
	5:1	0.387 ± 0.026	Weak
	10:1	0.357 ± 0.036	Weak
CD44	1:5	0.754 ± 0.055	Strong
	1:10	0.787 ± 0.039	Strong
	5:1	0.768 ± 0.077	Strong
	10:1	0.926 ± 0.015	Very Strong

**Table 10:** Pearson’s Correlation Coefficient (PCC) for stem cell markers in HMEC-1:CAF:BON cell studies

	Ratio HMEC:CAF: BON	PCC for BON:Marker	Interpretation
CD36	1:1:5	0.492 ± 0.121	Moderate
	1:1:10	0.681 ± 0.018	Moderate
	5:5:1	0.808 ± 0.021	Strong
	10:10:1	0.816 ± 0.002	Strong
ALDH1A1	1:1:5	0.151 ± 0.061	Weak
	1:1:10	0.229 ± 0.062	Weak
	5:5:1	0.270 ± 0.033	Weak
	10:10:1	0.245 ± 0.005	Weak

### Discussion

The studies described above combine both a gene and protein level analysis of cancer stem-like cell markers in two different co-culture systems: indirect and direct. The combination of these strategies allows for greater characterization of the cancer cells and stem markers. For instance, the BON cells demonstrated a small but significant increase (∼25%) in CD36 and ALDH1A1 at the gene level based on the indirect culture method at the 1:5 ratio but smaller effects at 1:10 (Figure 4). However, in the direct co-culture model there was no difference in CD36 between 1:5 and 1:10 sample groups, but there is a trend for higher levels of ALDH1A1 in the 1:10 group (Figure 5). Across all direct co-culture groups, the levels of ALDH1A1 expression seem to be lower than what is suggested by the indirect co-culture model. While different endothelial cell lines were used in these studies, the fact that dramatic differences are observed suggests that differential regulation of the same stem-like cell markers occurs based on different types of stimulation from endothelial cells. These results indicate that while gene expression levels may change due to paracrine factors, the ultimate expression of the markers in 3D TME-like models may be driven by other juxtracrine or mechanobiological factors. Therefore, furthering our understanding of cancer stem cell regulation depends on combining investigations at multiple levels to uncover the precise mechanisms that govern disease progression.

## Quantification and statistical analysis

All statistical analyses were performed by GraphPad Prism (Version 9, indirect co-culture) or Excel (Real Statistics Add-in, Release 7.6, direct co-culture). Data were analyzed by 2-sided t-tests with equal variance or ANOVA followed by post-hoc Bonferroni as appropriate (α = 0.05) by GraphPad (Prism). Triplicate experiments were performed. Quantitative data are presented as mean ± SEM. ∗p < 0.05, ∗∗p < 0.01, and ∗∗∗p < 0.001 are considered as statistically significant or very significant, respectively.

## Limitations

This protocol describes the two cancer cells for co-culture, which are breast cancer cells and pNET cells. For other cancer cells, this protocol may be applied, but it is recommended to perform pilot experiments and optimize cell numbers and experimental conditions. For slow growing cell types (doubling speed > 48 h) such as ECs including HUVECs, up to 200k cells per gel sample can be loaded. For fast growing cell types (doubling speed < 24 h), including aggressive cancer cell lines such as MDA-MB-231 or OVCAR-8, as few as 25k cells per gel sample can be loaded. If combining slow- and fast-growing cells, it is recommended to select ratios with fewer of the highly proliferative cells.

If cells are proliferating quickly in the gel, there will be several visible signs during culture. First, the gel will begin to become more opaque; this can be observed by eye or in a light microscope. Additionally, on the microscope, the gels may begin to collapse away from the PDMS rings as the cell density increases. This may be due to active matrix remodeling or contractile behaviors of the embedded cells.

## Troubleshooting

### Problem 1

PDMS rings are ripping during fabrication (Related to step 6).

### Potential solution

• The dimensions of the rings are important in both height (∼1 mm) and width (∼1 mm) for integrity during handling. Breakage may happen if the PDMS rings are not fully symmetrically punched during fabrication or if the sheets are cured on an uneven surface, and a “thin” region of the ring is created.
• The thinner the PDMS region on the ring, the more likely breakage is either during fabrication or handling.
• To address uneven heights, a bubble level can be used to check lab bench surfaces where PDMS curing is occurring.
• Moving the PDMS sheet to the 65°C oven may also improve the evenness of the sheet curing, as the process will happen faster before uncured PDMS may flow into an uneven sheet.

### Problem 2

PDMS rings detach from coverslips during handling or loading of gels (Related to steps 6, 27, and 28).

### Potential solution

• The attachment of PDMS rings to the coverslips is dependent on hydrophobic interactions from the silicone polymer and the glass surface. Usually this is sufficient to provide the necessary integrity for 10 mg/mL fibrin gels to polymerize in the timeline previously described.
• However, if you are piloting different gel formulations with other ECM proteins, any disruption of the PDMS/coverslip connection may cause leaking before the gel is fully set.
• An alternative fabrication technique may be used to create a physical bond between the PDMS and coverslip through the use of an oxygen plasma cleaner system. Briefly, treating both the PDMS and coverslips with plasma O_2_ will create a physical bond between the surfaces that ensures complete attachment.

### Problem 3

Bubbles form in gels during the ring assay set up (Related to step 27).

### Potential solution

• Preventing formation of bubbles is usually easier than removing bubbles.
  ○ To prevent formation, we recommend using a fast but gentle touch when dispensing cell-gel mixtures into the rings, pressing down only to the first stop of the pipette.
  ○ When swirling the solution to cover the full area, you should keep the dispensing button depressed to this first stop position.
  ○ If during loading, you are having trouble changing the pipette volume from 75 μL to 50 mL, we recommend preparing two pipettes: one set to 75 μL, another one set to 50 μL. Change the pipette immediately after you mix fibrinogen three times by 75 μL pipette.
• Bubbles may still be introduced, especially if the pipette is not well calibrated.
• If bubbles are present there are two options to try to remove them:
  ○ Positive Pressure/Rupture: take a second pipette with a clean, dry tip and quickly pipette up and down (with air) to cause the bubbles to rupture or burst.
  ○ Remove/Aspirate: take a second pipette with a clean, dry tip and quickly try to aspirate the bubble. Either of these techniques needs to be done quickly, to eliminate bubbles before the gel begins to set.

### Problem 4

Problems with gel stability or integrity during the experiment (Related to step 31).

### Potential solution

• The selection and optimization of cell concentrations is crucial to ensure measurable and observable differences between sample groups.
• Cells that proliferate quickly, including some cancer cell lines and fibroblasts, may degrade the fibrin matrix before the desired endpoint of the experiment. Users can determine if this is happening by checking in the microscope to visualize cell density within the gels, and checking to see if the gel remains fully in contact with the PDMS ring.
• As cells overgrow, the gel will become very dense and dark on microscope images, and the edges may pull away from the PDMS ring, indicating gel compaction. If this is occurring repeatedly, users may consider decreasing the concentration of cancer or stromal cells initially loaded. Lower limits we have tested for this parameter are 2.5 × 10^4^ cells per ring.
• If gel degradation or compaction occurs, it can prevent analysis via immunofluorescence by limiting staining efficiency or restricting additional cell growth in a manner that introduces artifacts to the system.

### Problem 5

Resulting fluorescence images from direct co-culture do not show significant or measurable signals (Related to steps 46 and 47).

### Potential solution

• Immunofluorescence staining of 3D microtissue systems can be more difficult to optimize compared to 2D staining.
• We recommend adding Tween-20 to all washes following permeabilization to help increase specific binding between antibodies and their targets, while diminishing non-specific binding to the ECM-based gels.
• Furthermore, if low signal is observed, we recommend increasing the concentrations of the primary antibodies of interest.
• However, if there is a high background or noise signal, we recommend increasing blocking time from 1 h up to overnight (maximum 12 h). The blocking solution may also need to be modified depending on the types and sources of antibodies used.
• Users should check manufacturer’s recommendations if this is necessary. Positive and negative controls should be tested for all antibodies to ensure the reagents are suitable for 3D immunofluorescence studies.

## Resource availability

### Lead contact

Further information and requests for resources and reagents should be directed to and will be fulfilled by the lead contact, Dr. Mary Kathryn Sewell-Loftin (mksewellloftin@uab.edu).

### Materials availability

This study did not generate any new unique reagents.

## Data Availability

Data sets for indirect co-culture method have been previously published.^2^ Full data sets including raw images used in co-culture studies described above are available upon reasonable request to the lead contact.

## References

[1] Sewell-Loftin M.K., Bayer S.V.H., Crist E., Hughes T., Joison S.M., Longmore G.D., George S.C. (2017). Cancer-associated fibroblasts support vascular growth through mechanical force. Sci. Rep..

[2] Guo Y., Jiang Y., Rose J.B., Nagaraju G.P., Jaskula-Sztul R., Hjelmeland A.B., Beck A.W., Chen H., Ren B. (2022). Protein kinase D1 signaling in cancer stem cells with epithelial-mesenchymal plasticity. Cells.

[3] Best B., Moran P., Ren B. (2018). VEGF/PKD-1 signaling mediates arteriogenic gene expression and angiogenic responses in reversible human microvascular endothelial cells with extended lifespan. Mol. Cell. Biochem..

[4] Alspach E., Flanagan K.C., Luo X., Ruhland M.K., Huang H., Pazolli E., Donlin M.J., Marsh T., Piwnica-Worms D., Monahan J. (2014). p38MAPK plays a crucial role in stromal-mediated tumorigenesis. Cancer Discov..

[5] Wang B., Chen Q., Cao Y., Ma X., Yin C., Jia Y., Zang A., Fan W. (2016). LGR5 is a gastric cancer stem cell marker associated with stemness and the EMT signature genes NANOG, NANOGP8, PRRX1, TWIST1, and BMI1. PLoS One.

[6] Schneider C.A., Rasband W.S., Eliceiri K.W. (2012). NIH Image to ImageJ: 25 years of image analysis. Nat. Methods.

[7] Forster, B., Van De Ville, D., Berent, J., Sage, D., and Unser, M. (2004). Extended Depth-of-Focus for Multi-Channel Microscopy Images: A Complex Wavelet Approach. In 2nd IEEE International Symposium on Biomedical Imaging: Nano to Macro (IEEE Cat No. 04EX821), Arlington, VA, USA, 1. pp. 660-663. doi: 10.1109/ISBI.2004.1398624.

[8] Zudaire E., Gambardella L., Kurcz C., Vermeren S. (2011). A computational tool for quantitative analysis of vascular networks. PLoS One.

[9] Bolte S., Cordelières F.P. (2006). A guided tour into subcellular colocalization analysis in light microscopy. J. Microsc..

