# Supplementary material for: Protocol for indirect and direct co-culture between human cancer cells and endothelial cells
Source: STAR Protoc. 2023 Apr 21;4(2):102177. doi: 10.1016/j.xpro.2023.102177 (PMC10160801; doi:10.1016/j.xpro.2023.102177)
Supplement: Document S1. Figure S1 and Tables S1 and S2 [file mmc1.pdf]

# Supplemental Information

(A)

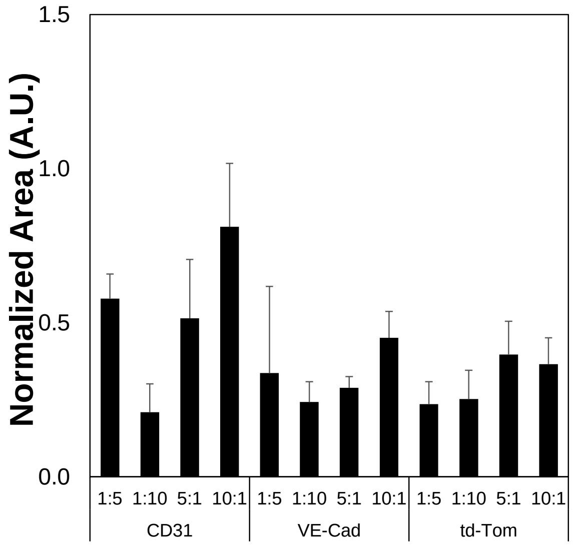


(B)

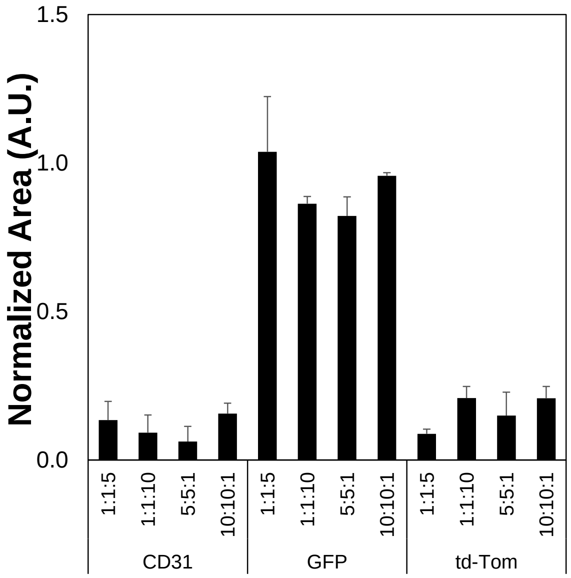


**Figure S1. Expression levels of ECs, BON cells, or CAFs in direct co-culture systems, related to steps 38-45 and 46-49.** Quantification of positive area for each stain for either ECs (CD31 or VE-Cad), BON (td-Tom), or CAFs (GFP) from direct co-culture studies, related to data shown in Figure 6. No significant differences were observed for any markers between cell ratio sample groups. GFP levels were generally higher than CD31 or td-Tom, which is most likely due to the increased proliferation rate of CAFs compared to the ECs and BON cells.

*Table S1. Pearson's Correlation Coefficient (PCC) controls for stem cell markers and in HMEC-1:BON cell studies, related to step 49 and Table 9*

| | **Ratio HMEC:BON** | **PCC** | **Interpretation** |
|---|---|---|---|
| **BON:CD31** | 1:5 | 0.311 ± 0.013 | Weak |
| | 1:10 | 0.119 ± 0.023 | Weak |
| | 5:1 | 0.308 ± 0.031 | Weak |
| | 10:1 | 0.315 ± 0.012 | Weak |
| **CD31:ALDH1A1** | 1:5 | 0.232 ± 0.010 | Weak |
| | 1:10 | 0.064 ± 0.008 | Negligibile |
| | 5:1 | 0.110 ± 0.001 | Weak |
| | 10:1 | 0.117 ± 0.010 | Weak |
| **CD31:CD36** | 1:5 | 0.281 ± 0.006 | Weak |
| | 1:10 | 0.095 ± 0.044 | Negligibile |
| | 5:1 | 0.228 ± 0.055 | Weak |
| | 10:1 | 0.261 ± 0.012 | Weak |
| **BON:VE-Cad** | 1:5 | 0.275 ± 0.026 | Weak |
| | 1:10 | 0.232 ± 0.041 | Weak |
| | 5:1 | 0.373 ± 0.027 | Weak |
| | 10:1 | 0.520 ± 0.069 | Moderate |
| **Ve-Cad:CD44** | 1:5 | 0.278 ± 0.016 | Weak |
| | 1:10 | 0.185 ± 0.043 | Weak |
| | 5:1 | 0.325 ± 0.044 | Weak |
| | 10:1 | 0.501 ± 0.085 | Moderate |

*Table S2. Pearson's Correlation Coefficient (PCC) for stem cell markers in HMEC-1:CAF:BON cell studies, related to Step 49 and Table 10*

| | Ratio HMEC:CAF:BON | PCC | Interpretation |
|---|---|---|---|
| **BON:CAF** | 1:1:5 | 0.200 ± 0.038 | Weak |
| | 1:1:10 | 0.096 ± 0.007 | Negligible |
| | 5:5:1 | 0.077 ± 0.013 | Negligible |
| | 10:10:1 | 0.113 ± 0.006 | Weak |
| **BON:CD31** | 1:1:5 | 0.288 ± 0.061 | Weak |
| | 1:1:10 | 0.236 ± 0.044 | Weak |
| | 5:5:1 | 0.149 ± 0.021 | Weak |
| | 10:10:1 | 0.238 ± 0.018 | Weak |
| **CAF:ALDH1A1** | 1:1:5 | 0.216 ± 0.106 | Weak |
| | 1:1:10 | 0.024 ± 0.008 | Negligible |
| | 5:5:1 | 0.020 ± 0.010 | Negligible |
| | 10:10:1 | 0.071 ± 0.003 | Negligible |
| **CAF:CD31** | 1:1:5 | 0.290 ± 0.016 | Weak |
| | 1:1:10 | 0.073 ± 0.005 | Negligible |
| | 5:5:1 | 0.048 ± 0.009 | Negligible |
| | 10:10:1 | 0.060 ± 0.017 | Negligible |
| **CAF:CD36** | 1:1:5 | 0.372 ± 0.033 | Weak |
| | 1:1:10 | 0.075 ± 0.020 | Negligible |
| | 5:5:1 | 0.028 ± 0.005 | Negligible |
| | 10:10:1 | 0.079 ± 0.007 | Negligible |

Note: Related to Step 49 and Table 9.
